# The effect of cytochalasin B – Loaded liposomes on the ultrastructure of the liver sieve

**DOI:** 10.1186/1476-5926-2-S1-S27

**Published:** 2004-01-14

**Authors:** Filip Braet, Katrien Vekemans, Henriette Morselt, Ronald De Zanger, Eddie Wisse, Gerrit Scherphof, Jan Kamps

**Affiliations:** 1 Laboratory for Cell Biology and Histology, Free University of Brussels (VUB), Laarbeeklaan 103, 1090 Brussels – Jette, Belgium; 2 Department of Cell Biology, Section Liposome Research, University of Groningen (UG), A. Deusinglaan 1, 9713 AV Groningen, The Netherlands; 3 Present address: Department for Molecular Biomedical Research, Molecular Cell Biology Unit, Ghent University (UGhent), Technologiepark 927, 9052 Zwijnaarde, Belgium

## Introduction

Liver sinusoidal endothelial cells (LSECs) possess fenestrae whose number can be increased both *in vitro *and *in situ *by depolymerizing the actin cytoskeleton [1]. Specially designed liposomes can be targeted with a high efficiency to LSECs. These liposomes, which were surface grafted with poly-anionized albumin (Aco-HSA) [2], can be filled with various substances, such as microfilament-disrupting drugs. This technique opens up attractive possibilities to modulate the liver sieve of LSECs with liposome-encapsulated microfilament-disrupting drugs *in vivo*.

The goal of this study was to alter the sieve's porosity by using cytochalasin B-loaded Aco-HSA liposomes. The increase in the liver sieve porosity induced by cytochalasin B (CB) may be exploited therapeutically to improve the extraction of atherogenic lipoproteins from the circulation; or to enhance the efficiency of liposome-mediated gene or drug delivery to hepatocytes.

## Methods

For *in vitro *studies, LSECs of the male Wistar rat were isolated by collagenase perfusion of the liver, isopycnic sedimentation in a two-step Percoll gradient, and selective adherence to different substrates. LSECs were cultured for 8 hours and treated with 0.025 microgram/ml CB or with Aco-HSA CB-loaded liposomes for 30, 60 and 120 minutes. In order to visualize filamentous actin (F-actin), LSECs grown on glass coverslips were stained with rhodamine-phalloidin [1]. Preparation of samples for EM-investigation and computer-assisted analysis was done according to standard protocols [1].

For *in vivo *experiments, male Wistar rats were injected with 2 micromolar (i.e., 0.45 ml) of liposomes via the penile vene. The liposomes were allowed to circulate for two hours. Control animals received free Aco-HSA, injected at the same concentration and volume. For all experiments, CB-loaded Aco-HSA liposomes with the following characteristics were used: 4.48 micromolar total lipid/ml; 47.6 micrograms Aco-HSA/micromolar total lipid; 0.32 microgram CB/micromolar total lipid; 0.025 microgram CB/ml.

## Results

**Figure 1:**
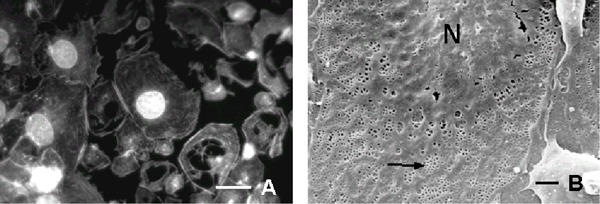
Fluorescence- and scanning electron micrographs of cultured LSECs treated with 0.025 microgram/ml CB for 2 hours (**A**) Staining of LSECs with rhodamine-phalloidin shows a loss of stress fibers. Bar = 15 –m. (**B**) Detailed topology investigation of CB-treated LSECs reveals intact fenestrae grouped in sieve plates (–). Note that a moderate increase in the number of fenestrae could be detected (for comparison see also, figure 3). Bar = 1 –m

**Figure 2:**
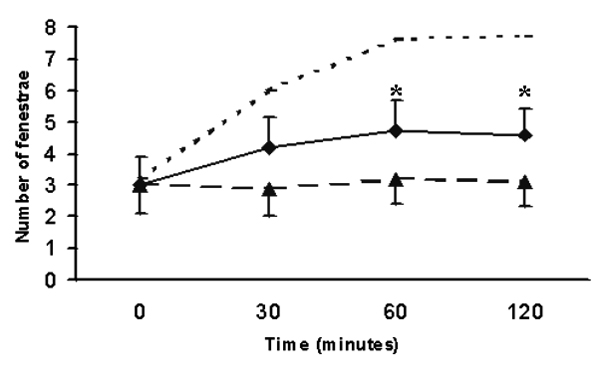
Effect of CB on the number of fenestrae per micrometer squared in time. Solid line shows the effect of 0.025 microgram/ml free CB on LSECs *in vitro *(* p &lt; 0.05; means – S.E.M); whereas the dashed line shows the effect of Aco-HSA CB-loaded liposomes *in vivo *at a final CB concentration of 0.025 microgram/ml. The pointed line above shows the expected effect of 10 micrograms/ml CB on LSECs *in vitro*.

F-actin staining showed a weak dissolution of cytoplasmic F-actin when cultured LSECs were incubated with 0.025 microgram/ml free CB (Fig. 1A). SEM-investigation of CB-treated LSECs showed a central lying nucleus and thin cytoplasmic extensions that contained clustered fenestrae in sieve plates (Fig. 1B). Computer-assisted analysis of endothelial fenestration showed a moderate but significant increase in the number of fenestrae per micrometer squared, i.e., from 3.1 – 0.4 to 4.3 – 0.3 respectively (Fig. 2).

**Figure 3:**
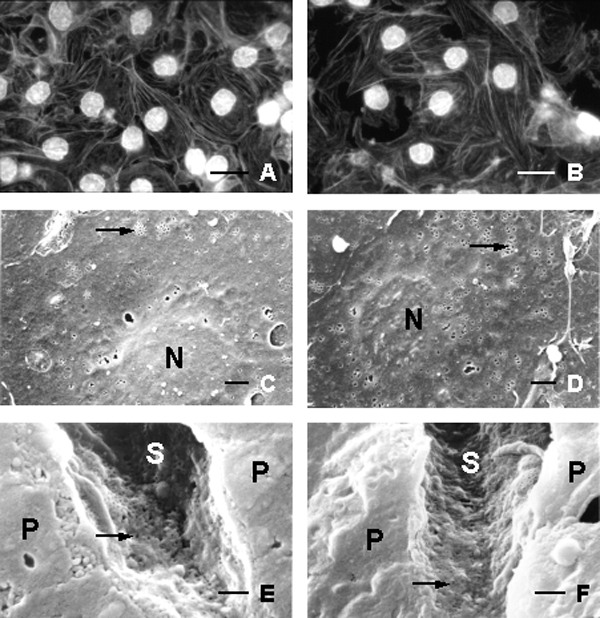
Fluorescence- and SEM images of LSECs *in vitro *(**A**-**D**) and *in vivo *(**E**-**F**) treated with Aco-HSA (**A**, **C**, **E**) (control) and with Aco-HSA CB-loaded liposomes (**B**, **D**, **F**) at a CB concentration of 0.025 microgram/ml for two hours. No signs of F-actin disruption could be observed in both conditions (**A**-**B**) (compare with Fig. 1A) and fenestral number remained unchanged (**C**-**D**) (compare with Fig. 1B). Nucleus (N), Fenestrae (arrow). (**E**-**F**) SEM images of the sinusoidal lumen (S) and parenchymal cells (P). Fenestrae (arrow). No significance difference in the number of fenestrae could be determined between the control (**E**) and rats injected with Aco-HSA CB-loaded liposomes (**F**). Bars A-B = 15 –m; C-D = 2 –m; E-F = 1 –m.

Examination of Aco-HSA treated cells or rats did not reveal any effects on the F-actin cytoskeleton (Fig. 3A) nor on the number of fenestrae per area (Fig. 3C) or on the ultrastructure of the liver sieve (Fig. 3E). When cultured LSECs (Fig. 3B,3C,3D) or rats (Fig. 3F) were exposed for 2 hours to 2 micromolar Aco-HSA CB-loaded liposomes, i.e., corresponding with 0.025 microgram/ml CB, no effect on the F-actin cytoskeleton or on the number of fenestrae per area could be detected (Fig. 2).

## Discussion

It is well known that CB disrupts F-actin in various cell types within 5 to 15 min after application. Previous studies on LSECs confirmed the rapidity of CB on the actin cytoskeleton and the number of fenestrae when relatively high doses of this drug were used, i.e., 10 micrograms/ml (21 micromolar/L). In this study, however, we used CB at a concentration of 0.025 microgram/ml and showed that this concentration induces a moderate but significant increase in the number of fenestrae *in vitro *(Figs. 1, 2). Unfortunately, due to technical limitations we were not able to load Aco-HSA liposomes with higher doses of CB, thereby excluding full comparison between the results obtained in this paper (Figs. 1,2,3) and the literature. Based on the data gathered, we suggest that there is a difference in cellular processing of free CB (Fig. 1) *versus *CB-loaded liposomes (Fig. 3). Aco-HSA-liposomes are taken up very efficiently by LSECs via a scavenger receptor mediated system [2]. Scavenger receptor mediated uptake is followed by efficient lysosomal degradation as was also shown for Aco-HSA liposomes. Therefore, we postulate that CB is not able to exert its effect anymore on the number of fenestrae after being in the lysosomal milieu. On the other hand, we have previously shown that CB is capable to inhibit the uptake of Aco-HSA liposomes in LSECs. The initial uptake of CB-loaded liposomes may inhibit further uptake of these liposomes, leaving the intracellular concentration of CB below the value that can be obtained with a free CB concentration of 0.025 microgram/ml. In summary, the obtained data show that targeted CB containing liposomes are not able to induce an increase in the number of fenestrae both *in vitro *and *in vivo*. This raises new questions regarding the uptake and processing of liposomes within cells.

## References

[B1] Braet F, Spector I, Shochet NR, Crews P, Higa T, Menu E, De Zanger R, Wisse E (2002). The new anti-actin agent dihydrohalichondramide reveals fenestrae-forming centers in hepatic endothelial cells. BMC Cell Biology.

[B2] Kamps JA, Morselt HW, Swart PJ, Meijer DK, Scherphof GL (1997). Massive targeting of liposomes, surface-modified with anionized albumins, to hepatic endothelial cells. Proc Natl Acad Sci.

